# Web-Based Structured Education for Type 2 Diabetes: Interdisciplinary User-Centered Design Approach

**DOI:** 10.2196/31567

**Published:** 2022-01-14

**Authors:** Shoba Poduval, Jamie Ross, Kingshuk Pal, Nikki Newhouse, Fiona Hamilton, Elizabeth Murray

**Affiliations:** 1 Research Department of Primary Care & Population Health University College London London United Kingdom; 2 Nuffield Department of Primary Care Health Sciences University of Oxford Oxford United Kingdom

**Keywords:** type 2 diabetes, patient self-management, diabetes education, primary care, digital health

## Abstract

**Background:**

Digital health research encompasses methods from human-computer interaction and health research.

**Objective:**

This paper aims to describe how these methods were combined to develop HeLP-Diabetes: Starting Out, a web-based structured education program for people newly diagnosed with type 2 diabetes.

**Methods:**

The development process consisted of three phases: initial design for effectiveness, optimization for usability, and *in the wild* testing in the National Health Service with people newly diagnosed with type 2 diabetes, and further revisions. We adopted an iterative user-centered approach and followed steps from the human-computer interaction design life cycle and the Medical Research Council guidelines on developing and evaluating complex interventions.

**Results:**

The initial design process resulted in an 8-session program containing information and behavior change techniques targeting weight loss, being more active, and taking medication. The usability testing was highlighted at an early stage, where changes needed to be made to the language and layout of the program. The *in the wild* testing provided data on uptake of and barriers to use. The study suggested low uptake and completion of the program, but those who used it seemed to benefit from it. The qualitative findings suggested that barriers to use included an expectation that the program would take too long. This informed refinements to the program.

**Conclusions:**

The use of interdisciplinary methods resulted in an iterative development process and refinements to the program that were based on user needs and data on uptake. The final intervention was more suitable for a definitive evaluation than the initial version. The description of our approach informs other digital health researchers on how to make interventions more sensitive to user needs.

## Introduction

### Interdisciplinary Research Methods

Research on digital health interventions (DHIs) brings together the human-computer interaction (HCI; which includes software engineering) and health (encompassing biomedical, behavioral, and social sciences). The research methods used in HCI, such as health research, are largely empirical (eg, experimental designs, surveys, and focus groups). However, health research tends to use a sequential approach, based on the methods used in pharmacological drug development, culminating in a randomized controlled trial to determine its effectiveness [1]. In HCI research, there is more emphasis on proximal (interaction) and distal (effects) outcomes, and the need to iteratively design and test an intervention until it is deemed to be *accessible and useful* by the user [2-4]. Acceptability and usability are crucial to digital health researchers, because the effectiveness of DHIs relies on being used (at the individual level), and the population impact depends on reaching a high proportion of the target population. The use of iterative methods common to HCI allows DHIs to be optimized until they are likely to achieve sufficient acceptability to ensure adequate reach, uptake, and use to achieve effectiveness and cost-effectiveness [5]. A decision about whether to proceed to a definitive randomized trial can then be made.

The Medical Research Council (MRC) has published guidelines for health researchers researching complex interventions to help them adopt appropriate methods. The 2006 MRC framework suggested a nonlinear approach to the development and evaluation of complex interventions, with four key stages: (1) development, (2) feasibility and piloting, (3) evaluation, and (4) implementation [6].

These 4 stages involve using evidence and theory to develop complex interventions, then testing them with a series of pilot studies aimed at key design uncertainties, before moving on to an exploratory and then a definitive evaluation [6]. MRC best practice guidelines is that definitive evaluation should only be undertaken once (1) the intervention and its delivery package reach a degree of stability, (2) any further development would be relatively minor, (3) there is reasonable confidence that the intervention could be implemented with high fidelity, and (4) there is a reasonable likelihood that the intervention will lead to improved health outcomes or equivalent outcomes at lower cost [5].

**Figure 1:**
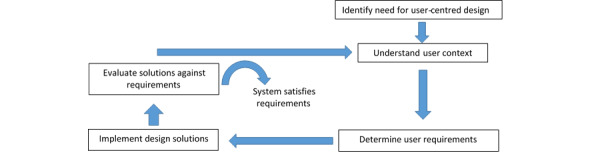
The human-computer interaction design cycle [10]. Used with permission from Elsevier.

Iterative development and evaluation are also features of the HCI development life cycles. A life cycle is the sequence of activities that occurs from the initial concept, through to the eventual phasing out and replacement [7]. The process purposefully cycles through several designs, incrementally improving the design until the final product is reached [7]. A key aspect is to be user-centered and involve users throughout the design process. This allows designers to understand people in the contexts in which they live, work, and learn, and consequently how to design products that fit easily into users’ everyday lives [8]. Standard frameworks for HCI and usability have been developed that recommend an iterative design process with an emphasis on the continuous identification of user requirements, testing the intervention against these requirements, respecifying user requirements, and retesting [9,10]. These processes inform each other and are repeated in each design cycle (Figure 1).

**Figure 2:**
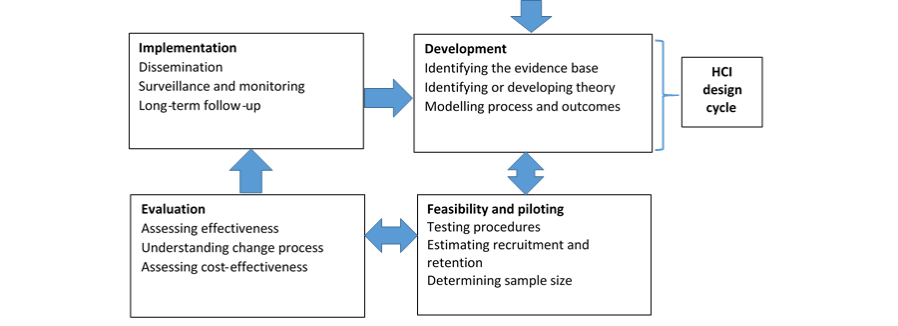
The Medical Research Council framework and human-computer interaction design cycle [1]. Used with permission from the BMJ Publishing Group Ltd. HCI: human-computer interaction.

The challenges of interdisciplinary work across HCI and health have been highlighted by Pagliari [11] and Blandford et al [2]. One of the key issues is that, although both the MRC and the HCI life cycle approaches are iterative, the HCI life cycle is located entirely in the development phase of the MRC framework, as illustrated in Figure 2.

This paper focuses on this development phase, with the aim of highlighting the importance of careful user-centered design and early-stage usability testing before further evaluation. We have reported how we tackled this, by describing the 3 phases of development and describing the methods from HCI and the methods from health that we incorporated at each phase. The aim of reporting our methods is to guide other researchers in developing similar interventions. Historically, complex interventions, such as diabetes self-management programs, have not been well described [12,13]. Better reporting would improve the understanding of causal mechanisms and increase the collective knowledge of how and why interventions work or not. This would, in turn, facilitate learning among researchers and the development of more effective interventions [14].

Our approach was also informed by guidelines on evaluating DHIs [5], which defines the research questions forming the basis of an evaluation of a DHI, and the issues that are particularly salient to DHIs rather than complex interventions as a whole. The guidelines on DHI evaluation was particularly relevant during the early stages of design when we needed to identify the health needs, target population, and causal model for the intervention.

There are other examples where the process of combining interdisciplinary methods have been documented [15-19]. The person-based approach developed by Yardley et al [15], for example, reports a development process involving qualitative interviews with a wide range of people from the target user population at every stage. Insights from users are then used to modify the intervention to make it more persuasive, feasible, and relevant. In keeping with the person-based approach, we collected insights from users and used these insights to modify the intervention at both the usability testing and *in the wild* testing stages.

### Background to the Intervention

Guidelines for evaluating DHIs recommend starting by defining the problem to be addressed, namely the health need that the DHI is intended to address and the population who could benefit from the DHI. For HeLP-Diabetes: Starting Out (HDSO), the health need that is being addressed is the provision of a structured education for people with type 2 diabetes mellitus (T2DM). T2DM is an international priority, affecting approximately 425 million people worldwide. T2DM places a considerable burden on patients in terms of premature morbidity and mortality and on health services, in terms of cost. Both these burdens can be reduced by structured self-management education, which can improve patient knowledge, self-care behaviors, metabolic control, psychological outcomes, and health care costs [20-23]. In the United Kingdom, T2DM affects an estimated 3.8 million people aged >16 years (8.6% of the population of this age group) [24] and accounts for approximately 10% of the total National Health Service (NHS) budget [25].

It is an NHS policy that all patients diagnosed with T2DM are offered structured education [26]. General practitioners (GPs) in England are remunerated through the Quality and Outcomes Framework (QOF) for referring newly diagnosed patients to suitable programs, with the suitability of the program determined by the accreditation by the Quality Institute for Self-Management Education (QISMET) [27,28]. Despite this incentivization, the uptake of structured education is poor (8.3% uptake in 2016 [29]). The reasons for this low uptake include difficulties with the current dominant model of structured education which is group-based and can be difficult for people who work, have caring responsibilities, or dislike groups. Our team had already developed a web-based self-management program (HeLP-Diabetes) [30], which was shown to be effective and cost-effective [31,32]. HeLP-Diabetes is a website with over 560 pages that provide self-management support for patients from diagnosis to death. The content is broken down into 8 sections, including information about understanding and treating diabetes, an interactive health record, news and research, and a forum and help page [31]. Engagement was also encouraged with regular emails and text that contained links to topical content within the website (eg, information regarding influenza vaccinations in winter) [30,33].

However, QOF payment and QISMET accreditation require a structured program (with a clear curriculum and learning goals and modules to work through in a linear fashion) aimed at newly diagnosed patients. HeLP-Diabetes was not structured (people have access to the website, without following a linear pathway), and it was not aimed at newly diagnosed patients but at patients at all stages of their diabetes journey. Therefore, we decided to develop a web-based structured course that could gain QISMET accreditation and meet the QOF requirements. The established courses that the GPs could refer patients to and gain QOF remuneration were all group-based and face-to-face; thus, a web-based structured course would provide an alternative that could potentially bypass some of the barriers to uptake described earlier. Table 1 illustrates the key differences between the HeLP-Diabetes website and the HDSO-structured course described in this paper.

**Table 1:** Key differences between HeLP-Diabetes and HeLP-Diabetes: Starting Out.

Feature	HeLP-Diabetes	HeLP-Diabetes: Starting Out
Target user	People with T2DM^a^ at any stage	Newly diagnosed people with T2DM
Size	8 sections, with 560 pages	5 sections, with selected content from HeLP-Diabetes
How the intervention was delivered	Nonlinear—people could access any part of the website and dip in and out as they pleased	Linear—people worked through modules one by one, and were given access to the next module once they completed the previous one
Curriculum	No curriculum—a wide breadth of information was available, and people could choose which topics to access depending on interest	Spiral curriculum—people worked through a series of modules and added to the knowledge they gained from previous modules in a *spiral* fashion

^a^T2DM: Type 2 diabetes mellitus.

The interdisciplinary development process required the skills of a multidisciplinary team of patients, GPs, diabetes nurse specialists, and health and HCI researchers. KP and JR formed part of the HeLP-Diabetes team, and KP and SP formed part of the HDSO team. EM led both teams.

### Aim and Objectives

This paper aims to describe how we combined the methods described in the MRC and HCI guidelines, using the development of the HDSO program as a worked example. The three stages of development we undertook were as follows: (1) phase 1—initial design, (2) phase 2—optimizing for usability, and (3) phase 3—*in the wild* testing and further revisions.

For each stage of development, we have described the methods from HCI, the methods from health, and how we combined the two. The evaluation of the final intervention for feasibility, acceptability, and impact is described elsewhere [34].

## Methods

### Phase 1: Design for Effectiveness

#### Methods From HCI: Establishing User Requirements for HeLP-Diabetes as a Precursor for HDSO

##### Focus Groups

The first steps of the HCI design process involve understanding the user context and requirements. This took place during the development of the HeLP-Diabetes website, before the development of the HDSO-structured program.

Understanding the contexts in which people live, work, and learn allows designers to develop products that fit easily into users’ everyday lives. Products that are easy to use are more likely to be acceptable to patients and more widely taken up. Extensive work in establishing the requirements of patients with T2DM went into the development of HeLP-Diabetes, the precursor to HDSO, and has been reported by Dack et al [30]. User requirements were conceptualized as features that would make people want to use the interventions (*wants*) and features needed to help improve health outcomes (*needs*). The HeLP-Diabetes team conducted focus groups with patients and health professionals (health professionals facilitated engagement with the program) to collect this information.

##### Usability Testing

The content identified as necessary in the focus groups was integrated by the design team and then reviewed by a participatory design group consisting of patients with T2DM. The content went through several iterations and was put through usability testing. Usability testing is commonly used in software engineering and HCI research. It has been described as “representative users attempting representative tasks in representative environments, on early prototypes or working versions of computer interfaces” [35]. Usability testing aims to find flaws in the interface that need improvement and to make products more sensitive to users’ needs at an early stage of development [36]. There is a wide range of techniques used in usability testing, including questionnaires, think-aloud observation, and interview-based techniques [37]. Usability testing for HeLP-Diabetes involved users *thinking aloud* while undertaking prespecified tasks (eg, finding specific information or using one of the self-monitoring tools), a technique common and unique to HCI [38]. Usability testing helped to optimize the navigation and interactive features of HeLP-Diabetes. Selected content from HeLP-Diabetes were used to develop the HDSO program, informed by evidence, theory, and modeling.

#### Methods From Health

##### Evidence

Systematic reviews of web-based diabetes self-management interventions [39-42] have found that the most effective components are (1) prompting of self-monitoring of behavioral outcomes, (2) provision of information on consequences of behavior, (3) barrier to identification or problem solving, (4) feedback on performance, and (5) interaction with health care professionals via the internet [39,40,42]. This evidence was combined with the theory regarding long-term condition self-management to determine the necessary components of the program. This theory is discussed in the next section.

##### Theory and Causal Modeling

The aim of the structured program was not only to impart knowledge but also to empower and encourage people newly diagnosed with T2DM to improve their self-efficacy (self-confidence in self-management) and emotional well-being by learning about living a healthy lifestyle, making the most of the NHS and staying motivated. There were many theories and theoretical models which related to the aims of the program. These included the Corbin and Strauss model for the work of living with a long-term condition [43] and behavior change theories. The Corbin and Strauss model was chosen because of its holistic approach to diabetes self-management and fit with education theory about multidimensional learning. Increasingly, learning has been construed as being multidimensional and involving the body, emotions, spirit, and the mind [44]. The Corbin and Strauss model for the work of living with a long-term condition also emphasizes the need to address the emotional aspects of disease and identity issues. Corbin and Strauss identified 3 sets of tasks involved in self-management [45] from qualitative work on the perception of patients about their long-term conditions. These are conceptualized as follows [43]:

1. Medical management: adopting healthy behaviors (eg, not smoking, exercising regularly, and eating healthy food), working with health professionals (eg, keeping appointments and following instructions), and taking medicines.
2. Emotional management: addressing the negative emotions associated with being diagnosed with a long-term condition.
3. Role management: coming to terms with the disruption to one’s sense of self, including adjusting to the *patient* role and managing the impact of one’s diagnosis on relationships with friends, family, and colleagues.

Behavior change theories were used because they can help predict how and when behavior change occurs [46]. Behavior change techniques (BCTs) are the strategies used in an intervention to promote behavior change [47]. They can be designed using behavior change theories. Interventions that use more theory-based BCTs have been found to have larger effect sizes compared with interventions that use fewer techniques in studies of digital health behavior change interventions [47].

**Figure 3:**
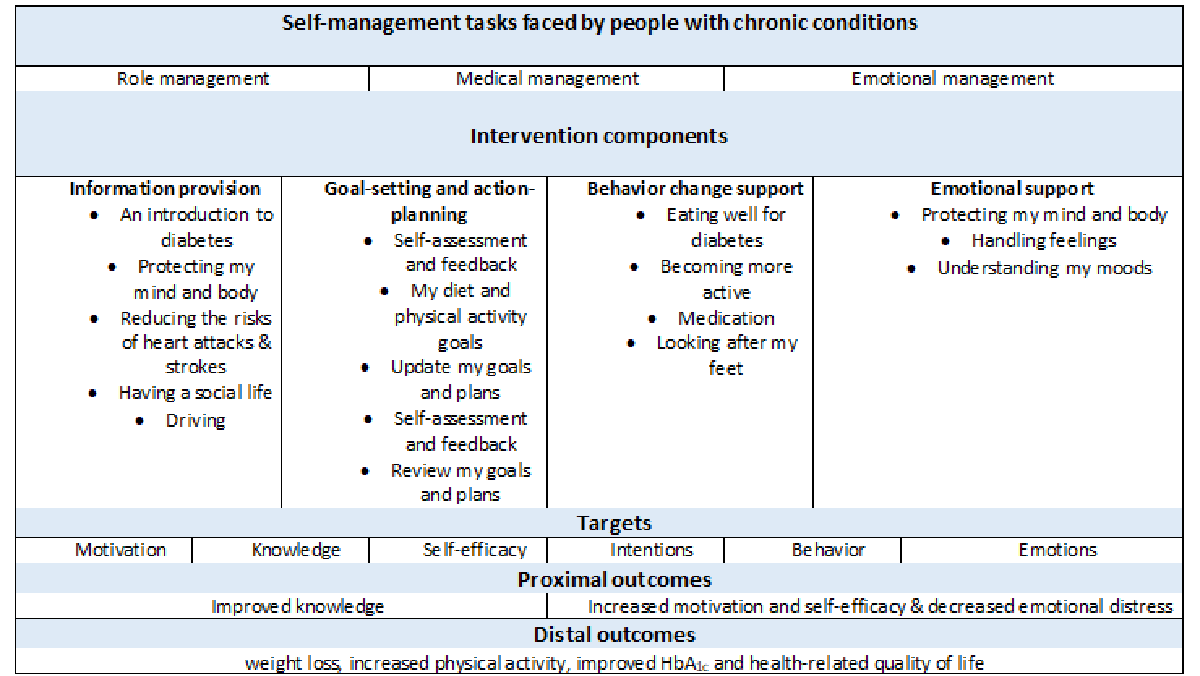
Causal model of HeLP-Diabetes: Starting Out program. HbA_1c_: glycated hemoglobin A_1c_.

Guidelines on evaluating DHIs [5] recommends identifying the necessary components of an intervention (including BCTs) by establishing a credible causal pathway for the intervention; thereby, linking evidence and theory to the intended outcomes. We linked the 3 self-management tasks identified in the Corbin and Strauss model to the intended outcomes of the intervention (improved knowledge, self-efficacy, and emotional well-being) using a causal modeling approach. The causal model for HDSO is illustrated in Figure 3.

In the long term, the proximal outcomes of knowledge, motivation, self-efficacy, and emotional distress combine to enable behavior change and better glycemic control. We opted not to measure long-term outcomes but focus on the short-term outcomes, including registration, use, and change in these 3 proximal outcomes.

##### How HCI and Health Methods Were Combined

Findings from the work on user requirements (HCI) and evidence, theory, and causal modeling of the intervention (health) were combined to select appropriate BCTs and develop the content, format, and structure of the program.

### Phase 2: Optimize for Usability

#### Methods From HCI

The usability testing of the HDSO program was conducted using questionnaires emailed to 5 patient volunteers. The questionnaires were written by the GPs and diabetes specialist nurses in the HDSO team and included the following four items: (1) the title of the session (eg, easy to understand and relevant to content), (2) the information contained in the session (eg, appropriate quantity of information, encouraging tone, and relevant links to information from other sources), (3) the visual design of the session (eg, readability of font, ease of finding access to videos, and the next section of the program), and (4) any other specific suggestions.

#### Methods From Health

Usability testing formed part of the early phase work recommended by the MRC and was undertaken to test the initial design with target users, before the exploratory *in the wild* testing with patients in the NHS.

#### How HCI and Health Methods Were Combined

Combining the phased approach advocated by the MRC and making changes to the program based on the results of 1 round of usability testing meant that the early development of the program was iterative. This was a key strength of this research. We did not immediately proceed to evaluation after the initial design of the program but undertook cycles of testing, refinement, retesting, and further refinement until we were more confident that the intervention fulfilled user needs and did not need major changes. This was recommended by both the MRC guidelines used by health researchers and the HCI lifecycle models used by HCI researchers. The refinements made to the design of the program were based on the results of the usability testing (and therefore based on user needs and experience).

The purpose of the next stage of testing and refinement was to evaluate the design of the program against user requirements. This was undertaken using *in the wild* testing.

### Phase 3: In the Wild Testing and Further Revisions

#### Methods From HCI

Research *in the wild* is a term used for research conducted in natural settings. It is increasingly used in HCI to understand how people react to and integrate technologies in their everyday lives over a period [48,49]. In situ studies are more likely to reveal the behaviors people adopt and the problems they encounter when they use an intervention at home, at work, or elsewhere. The advantage of this is that they provide greater external validity than experimental studies, where participants are more aware of how they are expected to behave. Another advantage is that *in the wild* studies nearly always provide unexpected findings regarding what humans do when confronted with a digital intervention; these can be the most informative findings [49]. In addition to usability testing, this provided an extra way of testing interventions against user requirements (as suggested in the HCI design cycle).

#### Methods From Health

The aim of the *in the wild* testing was to understand more about how patients were using the HDSO program in their everyday lives, including their experiences and views of the problems they encountered in using the program. Therefore, mixed methods were used in this study. Quantitative data were collected on the number of patients registering for and completing the program, patient characteristics, and changes in questionnaire scores. The questionnaires administered were the Problem Areas in Diabetes measuring diabetes-related distress [50], and the diabetes management self-efficacy questionnaire [51]. Questionnaires were included in the web-based program at the start and end of the course. Qualitative methods were used to explore the patient experiences and views regarding using the program.

Qualitative methods are used in both health and HCI research, but there are some important differences in the approaches used. For example, the *locus of expertise* differs. In health and social science research, researchers typically start with their own expertise rather than the user’s expertise, and the design interventions that (it is hoped that) users will engage with [2]. In HCI research, the user is assumed to be an expert in what they do and what they need. Digital health research has adopted more *user-centered* approaches to address the challenge of low uptake and adherence, with a focus on understanding and accommodating the perspectives of the people who will use the intervention [15]. In health and social science, a less formative (developmental) and more summative (cumulative) approach is often taken, so that there is less focus on early outcomes in the developmental stages and more focus on the impact of the final intervention. Emphasis is placed on conducting interviews of sufficient depth and duration. Interviews in HCI research use methods that are more common to industry and are driven by time and resources. Rapid user experience studies with smaller sample sizes are conducted at several stages during product development.

#### How HCI and Health Methods Were Combined

Methods from HCI and health were combined to conduct the *in the wild* testing of the HDSO program. The setting for the *in the wild* testing was GP practices. This was the natural setting for this study as referrals to structured education for T2DM patients occurs in primary care. Practices in 2 London boroughs that had taken part in a HeLP-Diabetes implementation study and practices in 2 London boroughs that were interested in commissioning the HDSO program participated in the study. The program was offered to these practices for free as an alternative to established face-to-face diabetes structured education courses that were already commissioned.

The study was submitted to the Health Research Authority (HRA) for NHS Research Ethics Committee (REC) for review. Secondary analysis of information collected as part of normal care was excluded from the REC review by the HRA as long as the patients were not identifiable [52]. Therefore, the collection of data on registrations, completed sessions, and questionnaire scores were permissible, as the data were automatically pseudonymized with a numerical identifier. Patients were informed on registration that anonymized data were collected by the program and used anonymously for ongoing service development.

A total of 15 practices agreed to offer the HDSO program to patients for the study. The program was offered to patients as an NHS service; therefore, there were no formal inclusion and exclusion criteria. Practices were informed that the target population of the intervention was adults (aged ≥18 years) with T2DM diagnosed in the last 9 months and asked to offer the program to everyone in this population. Practices were asked to identify eligible patients by running a search of the electronic medical records. Practices were sent registration packs to mail out to eligible patients. A total of 322 packs were mailed out. The registration pack contained information about the HDSO program (including that it was being offered as an NHS service as an alternative to face-to-face courses), how to register, and a reply slip. Patients interested in using the program returned a reply slip to the HDSO administrator with their contact details. Patients were then telephoned by the HDSO administrator who collected baseline demographic data (which was pseudonymized and added to the data collected automatically by the HDSO program) and created a username and password for the program. The HDSO administrator also confirmed whether they were happy to take part in research interviews and securely sent SP the ID numbers of all the patients who agreed. The username and password for the HDSO program were then emailed to the patient, along with information on who to contact if there were any problems.

We used qualitative telephone interviews with patients to explore their experience of using the program. We took an HCI approach of rapid data collection and used the data to inform optimization before further evaluation.

One of the members of the HDSO team (SP) contacted patients who registered for the HDSO program but did not start or complete it. We were unable to contact patients who did not register for the program, as they did not provide us with their details or consent to be contacted. The patients who did not start or complete the program were contacted, because we were particularly interested in the problems encountered with the program. The telephone calls were semistructured and lasted approximately 10 minutes. Questions included “What would help you to use the program more regularly?”

The interviews were carried out by telephone by SP, and written notes were taken rather than audio-recording and transcribing because the data needed to be collected and analyzed quickly to inform the program optimization. Note-taking is a recognized form of recording [53], and although it has the disadvantage of not capturing every word verbatim, the researcher mitigated this by noting down verbatim quotes where they were particularly pertinent.

### Ethical Approval

Ethical approval was obtained from the HRA (reference number: 159488). Data on registrations, completed sessions, and questionnaire scores were excluded from the HRA REC review, because of a clause that states that secondary analysis of information collected as part of normal care is excluded from REC review by the HRA, as long as the patients are not identifiable [52].

## Results

### Phase 1: Initial Intervention Components and Content

#### Establishing User Requirements for HeLP-Diabetes as a Precursor for HDSO

Results from the focus groups showed that patients needed help in managing the complexities of living with diabetes, such as managing the impact that irregular working hours had on diet and blood sugar, impact on relationships and social life, and support in dealing with the profound negative emotions caused by the diagnosis, which included anger, guilt, shame, and despair. The tools to help them manage these tasks included high quality, detailed information, personal stories from other people with similar experiences, and quizzes to test knowledge and provide feedback. Health professionals had similar perceptions of patient needs. Both patients and health professionals wanted HeLP-Diabetes to be interactive and visual (with quizzes, videos, and images), to be easy to use, and have a positive tone [30].

These results were combined with the results of the usability testing to create HeLP-Diabetes, a website containing 560 pages of information divided into 8 sections, which patients at any stage of their illness journey, could dip in and out of.

#### Content, Structure, and Format of HDSO

We used selected content from the HeLP-Diabetes website (informed by the causal modeling process) to construct the HDSO-structured program, which was needed to meet the QISMET and QOF requirements described in the introduction.

The causal modeling process helped us postulate that information and BCTs targeting healthy eating, weight loss, activity levels, smoking, alcohol consumption, and medication intake, would help users achieve the intended outcomes. Modules targeting these behaviors were therefore selected from the HeLP-Diabetes website to be integrated into the HDSO. The modules contained BCTs, including goal-setting, action-planning, self-monitoring, and feedback on performance [31]. These BCTs are based on the self-regulation theory [54], which states that our major self-regulative mechanism functions through (1) the self-monitoring of behavior, its determinants, and its effects; (2) the judgment of behavior concerning the person and place; and (3) effective self-reaction.

In addition to the BCTs from HeLP-Diabetes, self-assessment questionnaires and feedback were added as new components to the HDSO-structured course. The questionnaires assessed self-efficacy (self-confidence) in self-management, diabetes-related distress, and diabetes knowledge. These were positioned in the course in weeks 1 and 8 (before and after the program), thereby allowing users to reflect on the change in their scores.

Personalized emails were also added as new components to the HDSO-structured course. These were added to encourage motivation and engagement. A systematic review by Alkhaldi et al [55] on the effectiveness of prompts to increase digital interventions found that studies reported borderline small-to-moderate positive effects of technological strategies, including emails, to improve the use of interventions. Resource implications and mindfulness of our ultimate goal being HDSO delivered at scale across the NHS meant that emails were chosen as a cost and time-effective strategy for providing users with reminders.

A curriculum was needed to structure the content and components of the program and to achieve accreditation as a structured course. By identifying relevant theory (as suggested by the MRC guidelines), we decided that the program would follow a spiral curriculum based on the Harden and Stamper spiral curriculum model [56]. This model proposes that there should be an “iterative revisiting of topics, subjects or themes throughout the course.” The idea is that topics are not just repeated, but that knowledge and understanding should be deepened each time. The learner’s competence should increase with each visit until the overall aim is achieved [56].

The qualitative work that was conducted to establish user requirements for HeLP-Diabetes showed that users wanted information to be presented using text, images, and videos. These formats were therefore used to present information in the HDSO program and included videos of others living with diabetes. The text was written for people with a reading age of 12 to correspond with 80% of the population in the United Kingdom [57].

**Figure 4:**
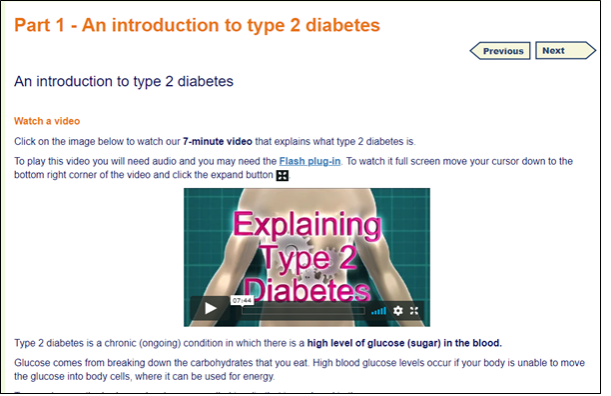
Screenshot of the HeLP-Diabetes: Starting Out program showing video component.

The result was an 8-session program containing information presented as text, images, and videos, and BCTs including goal-setting, action-planning, self-monitoring, and feedback on performance. Each session was designed to take approximately 40 to 50 minutes to complete and for people to complete 1 session per week. A screenshot of the HDSO program showing a video giving an introduction to T2DM is shown in Figure 4. The 8 sessions of the program and each of their parts are listed in Table 2.

**Table 2:** HeLP-Diabetes: Starting Out session titles and parts before usability testing and in the wild testing.

Session title and content		Session components
**Week 1—getting started**		
	Self-assessment	Self-assessment questionnaires
	An introduction to diabetes	Information
	Eating well for diabetes	Information
**Week 2—self-management**		
	Taking control	Information and quizzes
	Becoming more active	Information, physical activity goal-setting task, and videos of people’s stories
	Handling feelings	Information and videos of people with diabetes
**Week 3—improving my health and well-being**		
	Protecting my body and mind	Information
	Making changes	An exercise for reflecting on the quizzes in week 2, and setting SMART^a^ goals for diet, medication, activity, drinking, and other health behavior changes
	Understanding my moods	Videos of people with diabetes
	Working with diabetes	Information
**Week 4—taking control of my diabetes**		
	Making the most of the National Health Service	Information, videos of people talking about their interaction with the National Health Service, and a link to the health record in HeLP-Diabetes where users can record appointments
	Update my goals and plans	A review of SMART goals set in week 3
	Managing my moods	A reflection on the results of the mood quizzes in week 3, and a set of “Mood Tools,” including “Living Life to the Full,” a package developed by clinical psychologists using principles from cognitive behavioral therapy
	My social life	Information and videos of people’s stories
**Week 5—medication and lifestyle**		
	Medication	Information, videos about the challenges and benefits of medications, and an interactive “My medicines” list
	Review my goals and plans	A review and update of goals set in week 3
	How to fix almost everything	An opportunity to revisit the mood tools used in week 4
	Driving	Information
**Week 6—reducing my risks**		
	Reducing the risks of heart attack and strokes	Information
	Looking after my feet	Information
	Review my goals and plans	An opportunity to review and update SMART goals
	Living with diabetes	Videos of people talking about how they became used to having diabetes and an opportunity to revisit mood tools
**Week 7—working with my health care team**		
	Managing illness	Information
	My diabetes review	Videos about people’s experiences of diabetes care
	Review my goals and plans	Review and update SMART goals
**Week 8—celebrating success and planning for the future**		
	Self-assessment	Opportunity to repeat the self-assessment questionnaires from week 1
	Looking after my diabetes	Opportunity to prepare a care plan
	Moving on: the end of the beginning	Information about staying motivated and reading about diabetes in the media

^a^SMART: specific, measurable, achievable, realistic, and time bound.

### Phase 2: Usability Testing

Responses to the questionnaires described earlier were reviewed by the team. The relevant changes were made where there was agreement among responses from the patient volunteers. Where there was disagreement in responses from the patient volunteers, the suggested changes were discussed among the team members, and a consensus decision was made as to whether to make the changes. Changes were made to the language and layout of each session of the program, including making text and titles clearer and easier to understand for users.

Examples of questionnaire responses and changes made to the program, as a result, are given in Table 3, demonstrating how the progression of the development of the program was grounded in user needs.

**Table 3:** Example usability questionnaire responses and resulting design solutions.

Timeline	Questionnaire item	Response from patient volunteer	Design solutions
Week 1	Design	“Subtitles detailing content for each section would be helpful eg. Self-management 1 - Taking Control; Self-Management 2 - Getting Physical (Becoming more active); Self-management 3 - Handling Emotions (managing feelings?)”	Subtitles were added for each section of each module.
Week 2	Content	“First 3 pages good with 2 very useful video clips. Page 4 ‘Advice about increasing physical activity’ too much detail. Too many peoples stories at the end.”	Advice about physical activity was condensed, and the number of videos of people’s stories was reduced.
Week 3	Content	“Good, perhaps too much detail (contacts, addresses etc.) for sexual problems - could this be a website link? Level of detail might be off-putting for newly diagnosed.”	Contact details for support and advice organizations were removed and website links were added and signposted instead.

We made changes to the design and content of the program based on the questionnaire responses. This ensured that the development of the program was grounded in user needs. The changes included clearer subtitling, advice about physical activity made more concise, a more appropriate number of videos of people’s stories included, and links to support and advice organizations added.

### Phase 3: In the Wild Testing

During the study, 24 people registered for the HDSO program. Quantitative data were collected on program use, questionnaire scores, and characteristics. Of the 24 people registered, 3 (13%) people completed the program, 13 (54%) people started the program but did not complete it, and 8 (33%) people did not start the program. The data suggested low uptake and completion, but those who used it seemed to benefit from it (mean self-efficacy in self-management scores and diabetes knowledge scores increased).

The telephone interview responses were analyzed using a thematic analysis approach, and a list of barriers to completing the program emerged from the data. These are listed in Table 4 with illustrative quotes.

**Table 4:** Themes observed from the telephone interviews.

Theme	Illustrative quote
Lack of time to start or complete the program	“I’ve tried going through it during breaks at work, but I keep getting interrupted. I’ve only got to the ‘Welcome’ page.” “Can you give me an extra hour in the day?”
Expectation that completing the program would take too long	“It’s going to take a while, I need to be able to use it with a spare ten minutes.”
Ambivalence about starting	“It’s in the background, I keep it in mind.”
Feeling of content not being relevant to some users	“It’s not relevant to me, I don’t take medication.”

These themes were used to inform the refinement and optimization of the program as discussed in the next section. The ideas were followed up in subsequent interviews conducted as part of the evaluation of the final intervention and reported elsewhere [34].

### Design Solutions Resulting From the In the Wild Testing

The themes identified from the interviews suggested that there were patient and program factors which influenced program use. Patient factors such as ambivalence were difficult to address. However, we were able to shorten the program and provide users with quicker access to the program with web-based registration.

Following a discussion among the HDSO team, the following changes were agreed upon:

1. Reducing the number of sessions in the program: evidence from systematic reviews of engagement with digital behavior change interventions [58] and research on adult web-based learning [59] suggests that participants disengage if the intervention is perceived as too long or overly complicated. The decision about what content to retain and what to remove was made after discussions with the diabetes specialist nurses in the HDSO team who were trained educators and experienced in delivering face-to-face structured education courses. To determine what content to retain and what to remove, we discussed the data from the user experience interviews, reviewed the guidelines on T2DM management [26], and examined the curriculum closely. The 8 sessions were cut down to 4 sessions, with a fifth bonus session available at the end. Despite comments about its irrelevance to newly diagnosed patients, all aspects of the management of T2DM were retained because it was considered important to give people a good overview and understanding of the types of treatment they might receive in the future. Topics including *managing my diabetes when I’m ill, working with diabetes*, and *driving with diabetes* were taken out of the main course and moved to the fifth bonus session. The final 4-session intervention is described elsewhere [60] and contains the following sessions: *getting started, self-management, improving my health and well-being*, and *taking control of my diabetes*.
2. Reducing the number of questionnaires: we decided to reduce the number of questionnaires from 3 to 2 by removing the AdKnowl questionnaire. The AdKnowl (knowledge) questionnaire [61] was removed because it was significantly longer and more time-consuming than the other 2 questionnaires. Patient feedback suggested that they found the questionnaire burdensome and off-putting; the evidence we found from systematic reviews of diabetes self-management education programs suggests that there is a lack of a consistent positive relationship between knowledge and glycemic control and that factors other than knowledge are needed to achieve long-term behavior change [21]. Therefore, we prioritized the changes in distress and self-efficacy.
3. Web-based self-registration: it was decided to change to web-based registration to save time and to make it easier for patients to access the program quickly. The self-registration page included a demographic questionnaire, which allowed for the collection of baseline data. Telephone support from the HDSO team was still available for those who had difficulty registering on the web or using the program.

We decided to offer the program to everyone with T2DM and not just people who were newly diagnosed. The HDSO program was developed in line with the national clinical guidelines for GPs advising them to offer patients with T2DM structured education at and around the time of diagnosis [26]. However, we knew from the National Diabetes Audit that not all patients were offered structured education at the time of diagnosis, and of those who were offered it in 2016-2017, only 7.1% attended [29]. Therefore, many patients with T2DM who were not newly diagnosed have not received structured self-management education and are in need of it. In addition, data on the incidence and prevalence of T2DM in the United Kingdom show that T2DM prevalence rates have more than doubled between 2000 and 2013, but incidence rates have increased more slowly [62,63]. This suggests that there are more people being diagnosed younger and living longer rather than new diagnoses, which consequently suggests that it would be possible to recruit people who were not newly diagnosed to the HDSO program than people who were newly diagnosed. We decided to offer the program to everyone with T2DM and collect data on the duration since diagnosis. This allowed us to compare completion rates between newly diagnosed and non–newly diagnosed patients.

## Discussion

### Principal Findings

We have described the stages of the development of a web-based structured education program for people newly diagnosed with T2DM, HDSO. Methods from HCI and health research were used in combination at every stage. Methods from HCI put more emphasis on understanding user requirements and determining uptake. The *in the wild* testing allowed us to identify low completion rates, which were not picked up in the usability testing because it was conducted with highly motivated patient volunteers. The methods from health and the MRC framework for complex interventions emphasized the impact of the intervention. This helped us to understand the potential effectiveness of the intervention. The iterative development process that we went through with the intervention, in contrast to traditional piloting and feasibility studies conducted in health research [64], resulted in an intervention that was more stable and appropriate for a definitive trial than the earlier iteration. Proceeding to a trial too early can be problematic because trials do not detect whether the lack of intervention effect is due to implementation failure or genuine ineffectiveness [65]. Randomized controlled trials also fail to permit iterative improvements to the design and updates to technology [66].

### Comparison With Previous Work

The existing reporting of complex behavior change interventions is limited, and this prevents successful replication of successful interventions [14]. Reviews of web-based T2DM self-management interventions have reported extreme heterogeneity of interventions [67] and poor descriptions of the theoretical bases and active ingredients of the interventions [39]. This makes it difficult for researchers to identify and understand successful intervention components and to be able to design and implement successful interventions. The field of digital health research and web-based diabetes self-management is evolving rapidly, and it is important for future research that lessons can be learned from existing studies. This description of the development and content of HDSO helps add to the understanding of how and why web-based interventions for diabetes self-management (and other long-term conditions) work and can be used to inform future research in this area. In addition to describing the intervention, this study also adds to the understanding of how interdisciplinary methods from health and HCI can be used to develop a DHI. Previous studies by Blandford et al [2] and Pagliari [11] have described the challenges in using interdisciplinary research in the development and evaluation of DHIs, and this paper illustrates some of the concepts described in the literature using the example of a web-based diabetes self-management program.

### Strengths and Limitations

The strength of this research is the use of methods from both health and HCI. We combined the theory of living with long-term conditions and a user-centered design to understand and meet user requirements. This meant that users of different age groups, education levels, and ethnic backgrounds could use the intervention, as demonstrated by the analysis of the usage data from subsequent studies [68]. A weakness of the interdisciplinary approach in this study was the emphasis on time and resources when conducting qualitative interviews with program users. This meant that we conducted rapid user experience studies at several stages, with smaller sample sizes and limited depth and duration of interviews.

Another weakness of our approach was patient involvement. More extensive patient involvement could have been used in the design of the interview guide for the telephone interviews in the user experience study and data analysis. The patient volunteers could also have been asked to make their own suggestions for refinements to the first iteration of the program, instead of relying solely on the data.

### Conclusions

This paper describes how interdisciplinary methods can be used to develop a web-based structured education program for people newly diagnosed with T2DM. Methods were combined from human-computer research and health research. The reporting of the development processes for DHIs needs to continue, especially when interdisciplinary methods are used, for researchers to be able to learn from each other and create user-centered interventions.

## Abbreviations

BCT: behavior change technique
DHI: digital health intervention
GP: general practitioner
HCI: human-computer interaction
HDSO: HeLP-Diabetes: Starting Out
HRA: Health Research Authority
MRC: Medical Research Council
NHS: National Health Service
QISMET: Quality Institute for Self-Management Education
QOF: Quality and Outcomes Framework
REC: Research Ethics Committee
T2DM: type 2 diabetes mellitus
